# Histological and developmental insights into the herbivorous dentition of tapinocephalid therapsids

**DOI:** 10.1371/journal.pone.0223860

**Published:** 2019-10-30

**Authors:** Megan. R. Whitney, Christian A. Sidor

**Affiliations:** Department of Biology and Burke Museum, University of Washington, Seattle, Washington, United States of America; Monash University, AUSTRALIA

## Abstract

Tapinocephalids were one of the earliest therapsid clades to evolve herbivory. In acquiring derived tooth-to-tooth occlusion by means of an exaggerated heel and talon crown morphology, members of this family have long been considered herbivorous, yet little work has been done to describe their dentition. Given the early occurrence of this clade and their acquisition of a dentition with several derived features, tapinocephalids serve as an important clade in understanding adaptations to herbivory as well as macroevolutionary patterns of dental trait acquisition. Here we describe the histology of tapinocephalid jaws and incisors to assess adaptations to herbivory. Our results yield new dental characters for tapinocephalids including a peculiar enamel structure and reduced enamel deposition on the occlusal surface. These traits are convergent with other specialized herbivorous dentitions like those found in ornithischian dinosaurs and ungulates. Furthermore, these results demonstrate that while acquiring some specializations, tapinocephalids also retained plesiomorphic traits like alternate, continuous replacement. We interpret these findings as an example of how different combinations of traits can facilitate a derived and specialized dentition and then discuss their implications in the acquisition of a mammal-like dentition.

## Introduction

**Fig 1:**
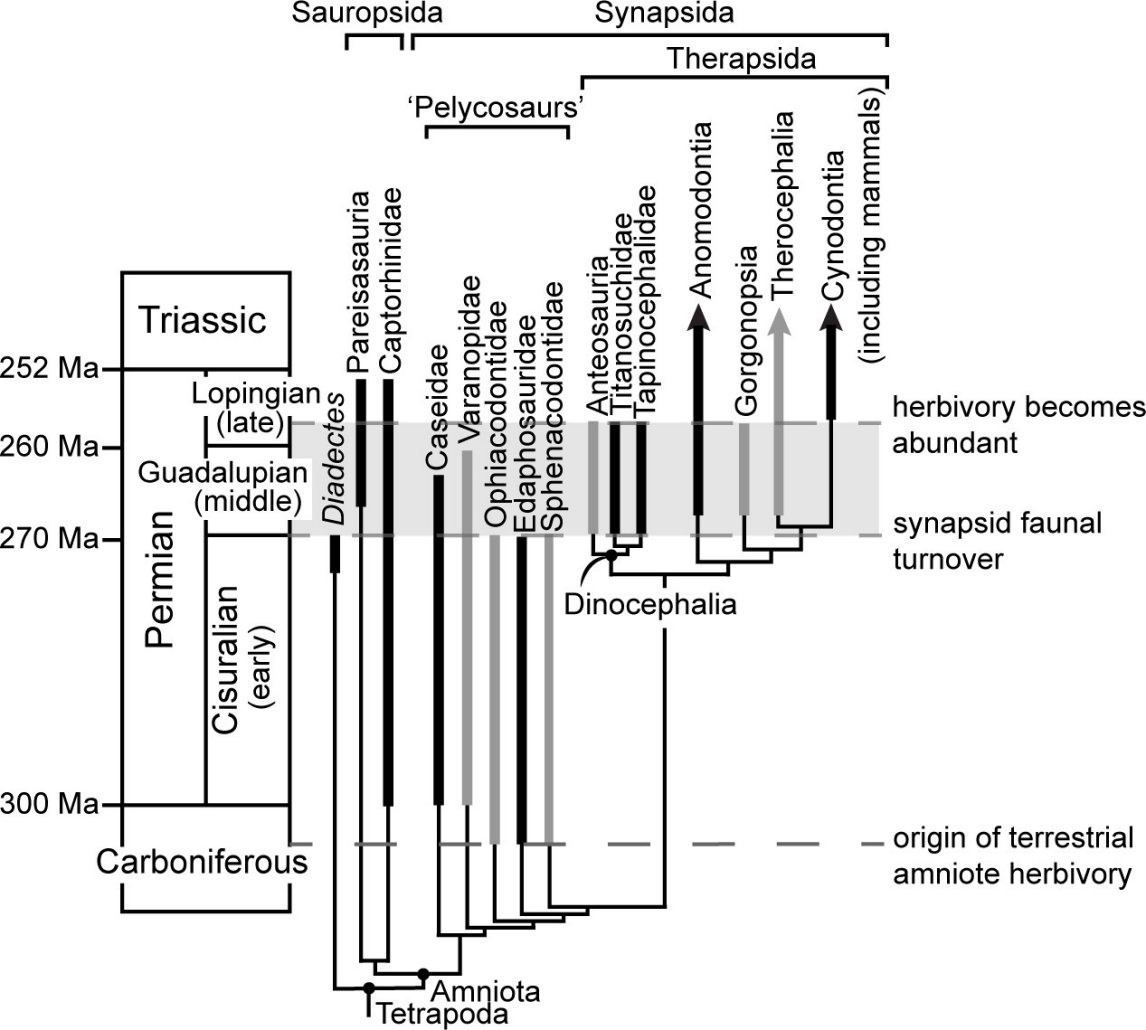
Simplified cladogram of Permian herbivores and major synapsid clades. The stratigraphic range of each taxon is represented by thick bars, with gray bar color characterizing carnivorous clades and black bar color signifying clades that include herbivorous taxa. The unique combination of trophic conditions (i.e. low abundance of terrestrial tetrapod herbivory and therapsid dominance) during the middle Permian is highlighted in gray.

Vertebrate herbivory is an integral feature of modern terrestrial ecosystems, evolving in sauropsid (reptile line), synapsid (mammal line), and early amniote lineages multiple times independently since its origin more than 300 million years ago in the late Carboniferous [1–3]. It was not until the late Permian (260 Ma), however, that herbivory became common among communities of terrestrial tetrapods [4]. Insects likely constituted most of the primary consumer trophic level during early to middle Permian times, although tetrapod herbivores were diversifying throughout that period [1–2, 5]. In addition to transitions on the trophic landscape, the middle Permian saw a synapsid faunal turnover resulting in a shift from a pelycosaur-dominated landscape to one dominated by therapsids [1, 5, 6] (Fig 1). This turnover was followed by diversification of therapsids, including several herbivorous clades and genera [1, 7]. These coinciding transitions give the middle Permian a unique set of trophic conditions in the history of vertebrate life on earth (Fig 1).

Synapsid herbivory in the early Permian was restricted to two clades of pelycosaur-grade synapsids, edaphosaurids and caseids (Fig 1), both of which show evidence of herbivory in their jaw musculature, barrel-shaped rib cage, and dentitions [4]. Their dentitions are similar to contemporaneous herbivorous sauropsids (i.e. captorhinids) with cutting or shearing crown morphologies and abundant palatal teeth [4]. By the middle Permian when therapsid synapsids began to dominate the landscape, edaphosaurids had gone extinct and only a single species of herbivorous caseid persisted [8]. The two clades with herbivorous therapsids during the middle Permian were the dinocephalians and anomodonts, both of which evolved dental specializations that differ from earlier occurring herbivorous amniotes.

Anomodonts have been recognized for their extremely derived dentitions and early instances of tooth-to-tooth occlusion, both of which have been related to efficient herbivorous processing [9–12]. Dicynodont anomodonts acquired a particularly derived dentition with a keratinized beak and often paired maxillary tusks that have been hypothesized for a variety of functions including foraging [13–15]. While anomodont adaptations to herbivory have been well documented [12–13, 16–17], dinocephalians have been comparatively overlooked by previous surveys of vertebrate herbivory during the Permian. Here we describe tapinocephalid dentition with special focus on microanatomical features that shed light on specializations acquired by these middle Permian therapsids.

### Background on tapinocephalids

Dinocephalian systematics has received relatively little attention in comparison to other therapsid clades like anomodonts, therocephalians, and non-mammalian cynodonts, but two major subgroups were recognized early on by Hopson and Barghusen [18]. Anteosaurs were large-bodied carnivorous dinocephalians known from southern Africa, China, Russia, and Brazil and recently Kammerer [19] has developed a robust phylogenetic framework for the group including two major subfamilies and nine genera. The other major dinocephalian subgroup is the herbivorous Tapinocephalia. Rubidge and van den Heever [20] described four tapinocephalian families including estemmenosuchids, stryacocephalids, titanosuchids, and tapinocephalids. The Russian estemmenosuchids and South African stryacocephalids are each known from one genus and their relative placement within Dinocephalia has been of historical debate [11, 21]. By contrast, titanosuchids, and to a greater degree, tapinocephalids contain several named taxa. Most tapinocephalian families have the plesiomorphic condition seen in anteosaurs and other therapsids more broadly, where an enlarged canine separates the jaw into incisal and postcanine regions [22]. The herbivorous dentitions of most tapinocephalians include incisors with a heel, pronounced canines, and bulbous (in Stryacocephalidae) or leaf-shaped (as in Estemmenosuchidae and Titanosuchidae) postcanines [20, 23–24].

**Fig 2:**
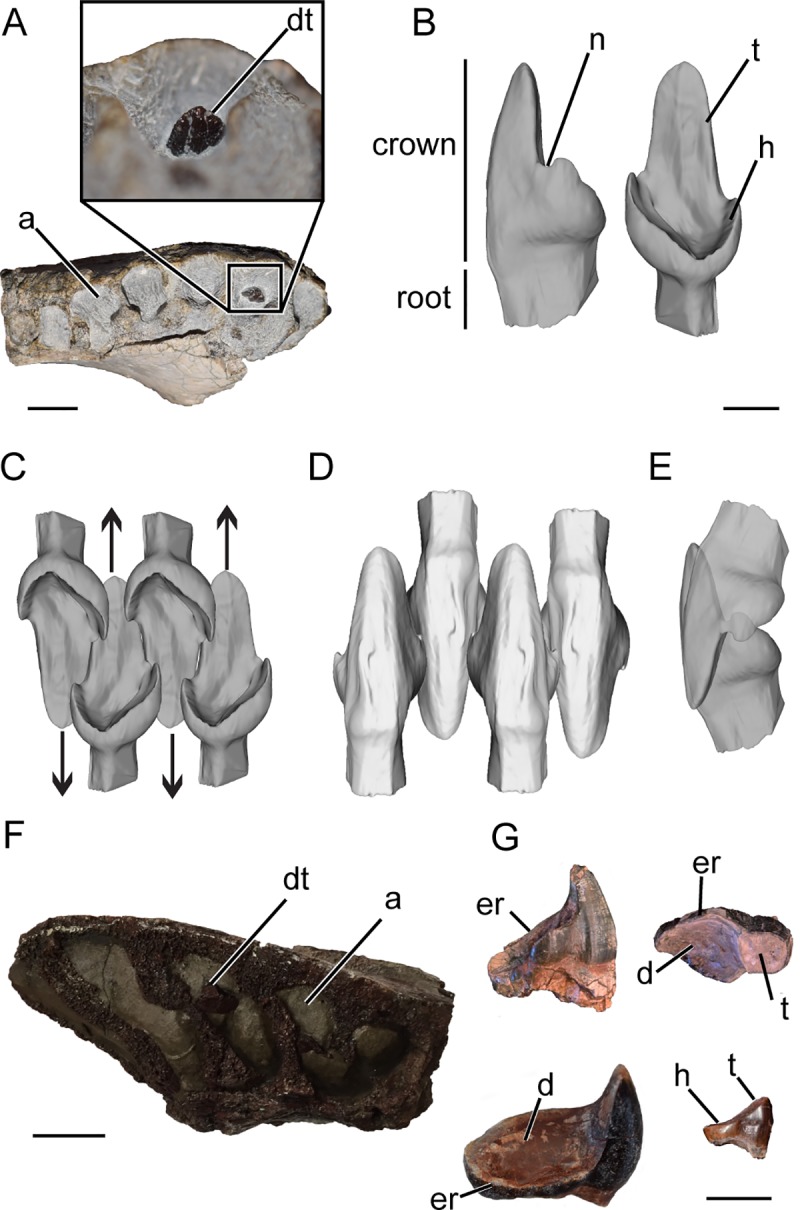
Tapinocephalid occlusion and dental morphology. A) Occlusal view of NHCC LB303 the anterior left dentary of a very small juvenile with a developing tooth (inset); scale bar = 1 cm. (B) Segmented μCT scan of the developing tooth of NHCC LB303 in distal (left) and lingual (right) views; scale bar = 0.5 cm. Model of tapinocephalid tooth-to-tooth occlusion using μCT scan and segmented tooth NHCC LB303 in (C) lingual, (D) labial, and (E) side lateral views. (F) Occlusal view of edentulous right anterior dentary of NHCC LB370 that contains a developing tooth. Anterior to left and labial up, scale bar = 3 cm. (G) Examples of Madumabisa Mudstone Formation tapinocephalid teeth with examples of heels comprised of enamel ridges and dentine basins. NHCC LB125 at top left in mesial view (assuming the tooth is from the lower jaw), NHCC LB131 at top right in occlusal view, NHCC LB126 at bottom left in occluso-mesial view to demonstrate wear on heel (assuming the tooth si from the lower jaw), NHCC LB1007 at bottom right in mesial view (assuming the tooth is from the lower jaw); scale bar = 2 cm. Abbreviations: **a**, alveolus; **d**, dentine; **dt**, developing tooth; **er,** enamel ridge; **h**, heel; **n**, notch; **t**, talon.

The noted exception to this generalized pattern is the most taxonomically diverse group of tapinocephalians, the tapinocephalids [23]. Tapinocephalids were a globally distributed, middle Permian group, with fossils known from Brazil, Russia, and southern Africa [7] and in contrast to other tapinocephalians, tapinocephalids are secondarily homodont with variation along the tooth row principally confined to a gradual reduction in crown size distally (e.g. Fig 2A). It is worth noting, however, that two potentially early diverging tapinocephalids, *Riebeeckosaurus* and *Tapinocaninus*, retain canines and *Riebeeckosaurus* possesses leaf-shaped postcanines similar to, albeit more bulbous than, other tapinocephalians [25].

The global distribution and peculiar shape of tapinocephalid teeth have been described in some detail [23, 25–30]. Tapinocephalid crowns retain the tapinocephalian incisor heel and talon morphology (Fig 2B) throughout the tooth row which facilitated precise tooth-to-tooth occlusion [23]. Tapinocephalids achieved precise occlusion by developing an elongated labial talon that intermeshes with the opposing and adjacent talons (Fig 2C–2E) and a labiolingually expanded heel for opposing upper and lower teeth [23] (Fig 2G). In addition to their dentition, further evidence of herbivory in the clade includes a large barrel-shaped rib cage [31] and aspects of the jaw adductor musculature that suggest a crushing bite force [32]. These gross anatomical features, however, do not address other possible adaptations to herbivory that can be revealed by microanatomical analyses. For instance, recent histological work has described a permanent ligamentous tooth attachment (i.e. a gomphosis) in tapinocephalids that would have provided shock-absorbing support during occlusion [33].

Given the early phylogenetic and temporal occurrence of herbivory in tapinocephalids and their acquisition of derived, once considered uniquely mammalian features (i.e. a gomphosis and precise tooth-to-tooth occlusion), further examination of their dentition can reveal additional derived characteristics and alternative combinations of traits that would have facilitated efficient oral food processing early in the evolution of synapsid herbivory. In addition to describing and adding details about tapinocephalid dentition, we detail interesting possible convergences with other specialized herbivorous dentitions (i.e. ornithischian dinosaurs and ungulates) and discuss possible implications for the combination of traits tapinocephalids acquired in a broader understanding of the macroevolution of dental characters.

## Methods

### Institutional abbreviations

**NHMUK**, Natural History Museum, London, United Kingdom; **BP,** Evolutionary Studies Institute (formerly Bernard Price Institute), University of Witwatersrand, Johannesburg, South Africa; **NHCC**, National Heritage Conservation Commission, Lusaka, Zambia; **SAM**, Iziko:South African Museum, Cape Town, South Africa.

### Specimen collection and curation

All necessary permits were obtained for the described study which complied with all relevant regulations. Excavation, temporary export, and research permits for Zambian fossils were obtained from the NHCC (Lusaka). Locality information is available to qualified researchers. Comparative material was examined with permission of the relevant curators at the NHMUK, Evolutionary Studies Institute, SAM, the Field Museum of Natural History, and the American Museum of Natural History.

### Specimen selection and identification

Specimens selected for this study include fragmentary dentaries and isolated teeth from the Madumabisa Mudstone Formation of the Mid-Zambezi Basin of southern Zambia [29]. Isolated teeth included in this study measured between 21–37 mm in labiolingual width and had heels with exaggerated labiolingual extensions (i.e. heel length: mesiodistal width > 1), comparable to those reported from similar localities [29–30]. Isolated teeth that did not show these proportions (i.e. heel length: mesiodistal width < 1) were not used as to avoid the inadvertent inclusion of titanosuchian incisors, which can be morphologically similar to more posterior tapinocephalid teeth. Dentaries were identified as belonging to tapinocephalids by their relatively large size compared to other vertebrate fossils from localities, the lack of pronounced canine alveoli [23], and oftentimes the lack of in situ teeth, which has been a noted feature of this clade (e.g. Fig 2A and 2F) [21, 23, 29].

In addition to these adult or sub-adult specimens, we included a very small partial left dentary, NHCC LB303, that provides data on early ontogeny in tapinocephalid dentition (Fig 2A). To our knowledge, this specimen represents the smallest tapinocephalid individual known and thus likely a very young juvenile. The dentary includes the symphysis as well as the first seven tooth positions, which measures approximately 54 mm long. We estimate the total jaw length to have been 100–130 mm when complete. NHCC LB303 can be assigned to Tapinocephalidae primarily because of the gradation in tooth socket size moving mesially to distally (i.e. no indication of heterodonty, lack of an enlarged canine tooth position) [23]. Furthermore, the contribution of the splenial to the mandibular symphysis has been described in some titanosuchians [34] which is not apparent in NHCC LB303 where the symphysis is almost entirely composed of the dentary. Unfortunately, this specimen fails to preserve several anatomical features that have noted variation in tapinocephalids throughout ontogeny (e.g. cranial pachyostosis, [35]), which could provide alternative evidence of its ontogenetic stage.

The dentary of NHCC LB303 is edentulous, like most tapinocephalid fossils, but preserves a single nearly completely developed incisor that was close to eruption (Fig 2A). Micro-computed tomographic (μCT) scans of the specimen reveal that root formation was incomplete, but most of the crown development, including enamel deposition, was complete. The incisor has a well-developed heel and talon with a notch at the junction between heel and talon which is visible in lateral view (Fig 2B). The ratio of heel length: mesiodistal width (0.65) and presence of a notch is most similar to proportions observed in the isolated tapinocephalid teeth from specimen SAM-PK-323 (0.55) and BP/1/7287 (0.52), *Moschops* specimen AMNH 555 (0.53), and *Struthiocephalus* specimen SAM-PK-115911 (0.53). Interestingly, this is in contrast to the vast majority of isolated Zambian tapinocephalid incisors that are consistently at a ratio > 1 (e.g. Fig 2G). Due to the lack of material associated with NHCC LB303 as well as a dearth of characters diagnostic to genera within Tapinocephalidae, we are unable to assign this specimen to a genus.

### Microanatomical analyses

Of the specimens selected for microanatomical analyses, five were thin sectioned, two were scanned under an electron microscope, and one was scanned using μCT (Table 1). Histological sectioning was conducted following the established methods described by Lamm [36] and quantitative and qualitative data were collected under a Nikon Eclipse POL100 polarized microscope in plain and cross-polarized light using NIS Elements software. Scanning electron microscopy (SEM) was used to examine enamel microstructure. Epoxy embedded specimens were surface etched with 10% HCl and gold sputter-coated images were collected using JEOL JSM-6610LV and carbon-coated images with JEOL JSM 7000F scanning electron microscopes. μCT images were collected using NSI X5000 scanner at 2K resolution with a 24μm voxel size.

**Table 1:** Specimens included in this study and analyses performed.

Specimen #	Element	Analyses
NHCC LB653	Talon portion of tooth	Histological, SEM
NHCC LB733	Tooth with partial root	Histological, SEM
NHCC LB298	Ejected tooth with resorbed root	Histological
NHCC LB369	Partial edentulous dentary	Histological
NHCC LB303	Anterior left dentary	μCT
NHCC LB370	Right edentulous dentary in two parts: Incisal region with erupting tooth Postcanine region with developing tooth	Histological Histological

### Frequency and pattern of tooth replacement

The rate of tooth replacement, although not an exact measure of the number of replacement events through the lifetime of an animal, has been estimated in fossil taxa, mostly dinosaurs (e.g. [37–38]). These studies have employed methods developed by Erickson [39] that examine the difference in daily incremental growth lines between a functional tooth and its developing replacement.

However, given our current sample of tapinocephalid teeth, employing the replacement frequency techniques established by Erickson [39] would be unreliable. The edentulous nature of most tapinocephalid jaws, especially those available for thin sectioning in this study, render the sample such that none of the jaws had a developing tooth and its functional predecessor in the same position. Furthermore, the lack of rigorous ground-truthing for the effects of phylogeny or ontogeny on dentine growth make approximations in synapsids precarious.

To develop an alternative estimate for replacement frequency, we collected data on the proportion of teeth in a jaw at different stages of replacement in 31 tapinocephalid jaws as well as several other non-mammalian synapsids for comparison (92 pelycosaurs-grade synapsids, 23 other dinocephalians, 27 gorgonopsians, 20 therocephalians, 23 non-mammalian cynodonts). Although not a direct measure of replacement rate, these data provide generalizations about the relative rate of replacement in tapinocephalids compared to other fossil synapsids. For each jaw examined, we categorized each tooth position in one of the following four categories: 1) functional tooth present, 2) empty alveolus without preservation of the developing tooth, 3) replacement tooth present in its developing alveolus (i.e. crypt), and 4) replacement tooth in functional position but not fully erupted. All three latter categories were considered replacement events and compared to the total number of tooth positions in the jaw. Smaller, presumably sub-adult and juvenile specimens (e.g. NHCC LB303) were excluded because it is likely that they were replacing their teeth at a more rapid rate than adults, possibly making standardization across various groups of synapsids inconsistent.

## Results

### Dentine

**Fig 3:**
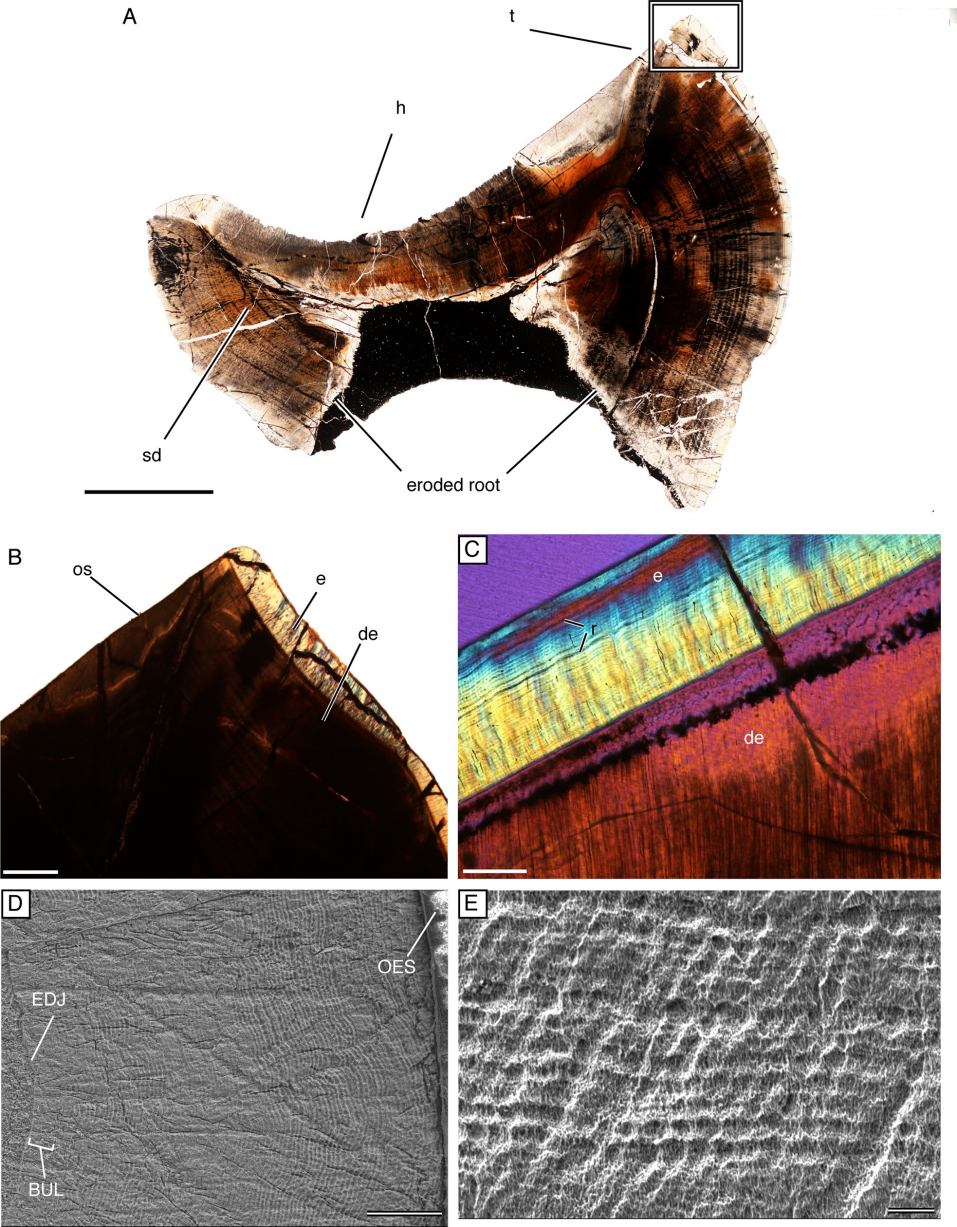
Enamel microstructure of tapinocephalid teeth. (A) Longitudinal thin section of NHCC LB298 that was ejected from the jaw as evidenced by the eroded root and secondary dentine. Labial to the right and occlusal to the top of the page; scale = 5000 μm. (B) Histology under cross polarized light of the tip of the talon of NHCC LB298 with thick enamel on the labial edge and no enamel on the lingual, occlusal surface; scale = 1000μm. (C) Under cross polarized light and a lambda plate, a section of NHCC LB298 enamel with incremental lines of Retzius; scale = 100μm. (D) SEM image of NHCC LB653 with relatively uniform prismless, coarse wavy-like enamel from the DEJ to OEJ; scale = 100μm. (E) Close up SEM image of NHCC LB653 with coarse wavy-like enamel in helical arrangement; scale = 10μm. Abbreviations: **BUL**, Basal Unit Layer; **de**, dentine; **e**, enamel; **EDJ**, enamel-dentine junction; **h**, heel; **OES**, outer-enamel surface; **os**, occlusal surface; **r**, striae of Retzius; **sd**, secondary dentine; **t**, talon.

All sectioned teeth have thick dentine walls with dentine tubules and periodic depositional growth marks of both small-and large-scale increments. Mostly primary dentine was observed, with secondary dentine visible in a shed incisor (NHCC LB298; Fig 3A), confirming that this tooth had completed root formation and was in occlusion [40]. The amount of dentine deposited between incremental lines varied between 20 and 40μm and averaged at about 34μm per depositional period, similar to the 5–10 day cycles seen in mammals [41–42].

### Enamel

Very little has been published on the enamel of tapinocephalids, although Sidor et al. [29] noted the presence of wrinkled enamel similar what is seen in sauropod dinosaurs. Enamel caps tapinocephalid teeth except for the heel where dentine is exposed (Fig 3B), either due to wear or possibly congenitally (see *Development*). Enamel thickness varies along the long axis of the tooth with the thickest enamel observed mid-crown (approximately 0.4–0.59 mm) and tapering towards the cervical (i.e. towards the root) and occlusal ends (approximately 0.3–0.1 mm). There is also a slight difference in enamel thickness between the lingual and labial edges of the heel, with thicker enamel labially (approximately 0.4–0.5 mid-crown) than lingually (approximately 0.1–0.3 mm mid-crown) The enamel shows no evidence of wear on these surfaces, indicating that the variation in thickness between the labial and lingual sides of the teeth is a result of differential deposition. In thin section, regular incremental lines are visible in the enamel with a distance of about 6.5 μm between each line and as many as 60 lines at the thickest point (Fig 3C). We interpret these incremental lines as striae of Retzius, given their similarity to structures seen in other non-mammalian synapsids [43].

SEM imaging revealed a lack of organizational units (i.e. columns or prisms) in the crystallite structures of tapinocephalid enamel (Fig 3D and 3E). Instead, tapinocephalid enamel appears most similar to what has been described as coarse wavy enamel by Sander [44–45], Hwang [46], and Chen et al. [47] with crystallites set at oblique angles relative to the dentine-enamel junction (DEJ) and gradual changes in angles towards the outer enamel surface (OEJ) (Fig 3E). Although SEM imagery reveals similar structure to what has been described as wavy enamel, the distinct wavy pattern observed by Hwang [46] and Chen et al. [47] under cross-polarized light does not appear in the tapinocephalid material sampled here. SEM data suggest a relatively uniform crystallite structure throughout tapinocephalid enamel, with the exception of the thick Basal Unit Layer (Fig 3D), which is approximately 30 μm thick and made of finer wavy structural units.

### Crown morphology and development

**Fig 4:**
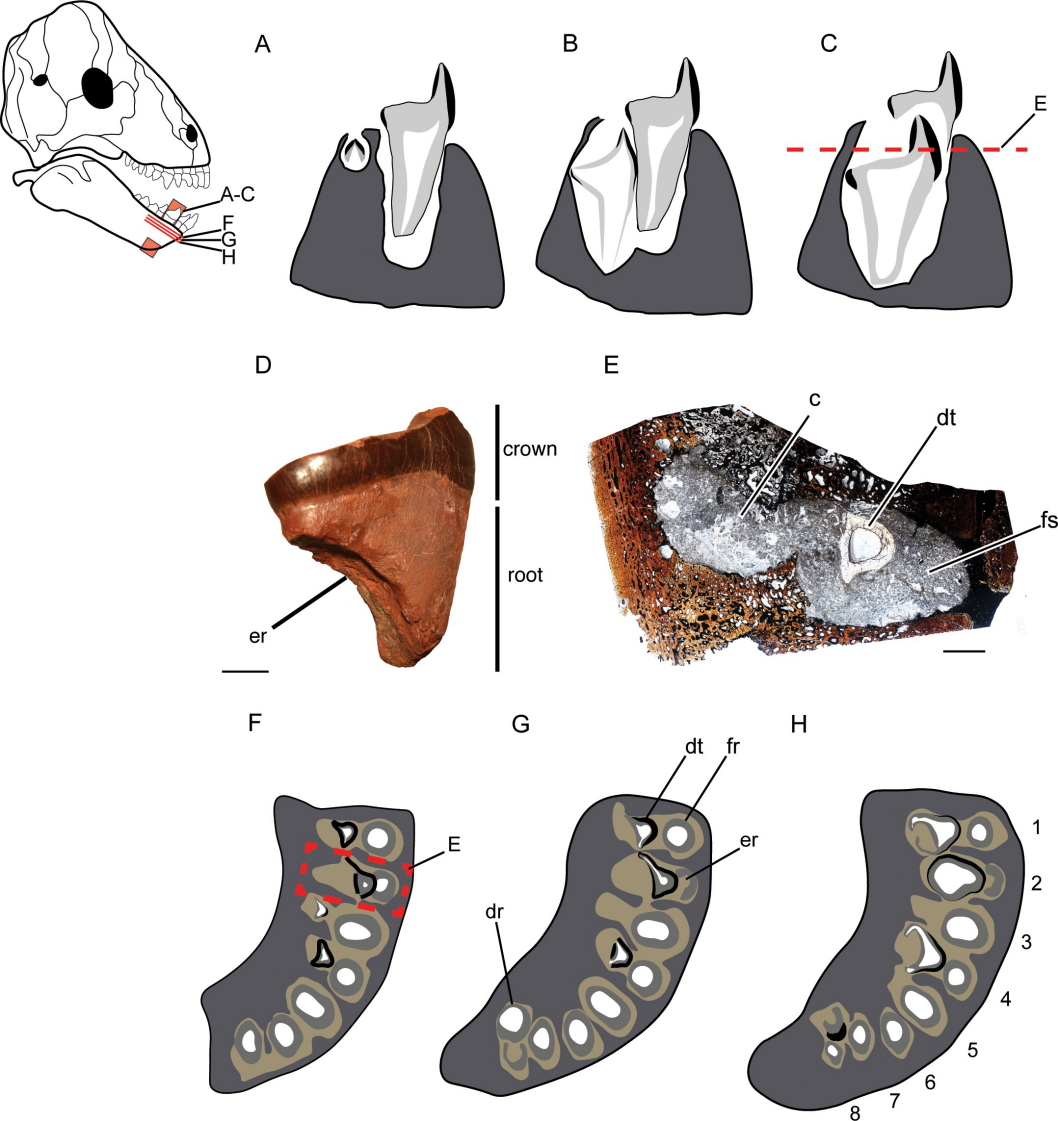
Tapinocephalid tooth development and replacement. All images are oriented lingual to the left and for A–D, occlusal is to the top of the page. (A) Bell stage development in a crypt lingual to functional tooth. (B) Crown formation and joining of the crypt and functional socket. (C) Erosion of the function tooth and movement of the developing tooth into the functional position. (D) NHCC LB827 was in the process of being replaced as evidenced by the lingual erosion of its root. Scale = 1 cm. (E) Histology of NHCC LB369 in horizontal section, with the talon of a developing tooth moving into the functional position. Scale = 5000μm. (F-H) Occlusal (F) to apical (H) schematics of sections taken of SAM-PK-12139 demonstrating different developmental stages of replacement as well as the alternating replacement pattern. Enamel is black and dentine is gray in A–C and F–H. Abbreviations: **c**, crypt; **dr**, developing root; **dt**, developing tooth; **er**, eroded root; **fr**, functional root; **fs**, functional socket.

Thin sections of the jaws of tapinocephalids capture various stages of crown development providing new data on the underlying processes giving rise to the peculiar morphology. Our data suggest that the complex morphology of a heel and talon differs developmentally from mammalian crown formation. In mammals, complex crown shape is initiated prior to mineralized tissue deposition such that the shape of the interface between the inner enamel epithelium and papilla (where deposition will begin) corresponds to the future outline of cusps with multiple enamel knots [48]. Tapinocephalids initiate tissue deposition of both enamel and dentine in a standard bell shape formation contained within a crypt lingual to the functional tooth (Fig 4A). None of our thin-sections provide evidence of additional enamel knots developing to form the heel and thus, we propose that this initial bell stage deposition continues cervically to create the talon and subsequently extends lingually and cervically to form the heel (Fig 4B). The resorptive crypt of the developing tooth eventually meets the alveolus of the functional tooth during development with the developing tooth moving into its functional position (Fig 4C). Sidor et al. [29] and Simon et al. [30] previously noted a semicircular lingual erosion of the roots of isolated tapinocephalid teeth and suggested that this erosion was likely the result of a developing tooth initiating root resorption (e.g. Fig 4D). Coronal and sagittal thin sections of developing teeth as well as transverse sections of alveoli confirm this proposal: merging of the functional alveolus and developing crypt happens well before ejection of the functional tooth (Fig 4E). This is further evidenced by serial sections taken through a *Tapinocephalus* dentary (SAM-PK-12139) that show merging of the two alveoli and erosion of the functional tooth root (Fig 4F–4H).

**Fig 5:**
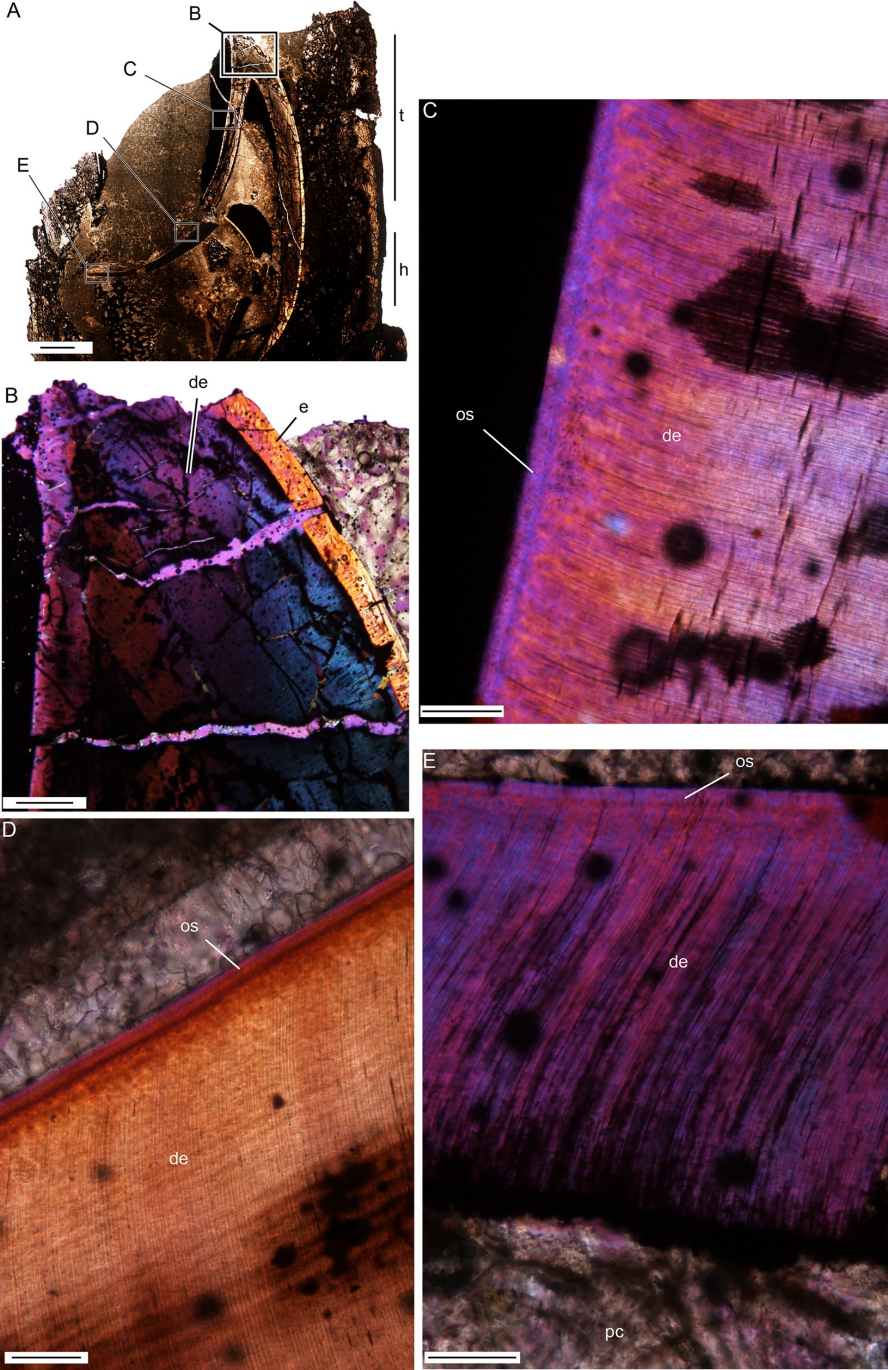
Developing tooth in the second incisor position of NHCC LB370 with no enamel deposition along the occlusal surface. (A) Sagittal section through the tooth demonstrating heel and talon development, with locations of subsequent images noted. Scale = 5000μm. (B) Tip of the preserved talon with substantial enamel deposition labially and none lingually. Scale = 1000μm. (C-E) The dentine and occlusal surface along the talon (C), labial portion of the heel (D), and lingual most portion of the heel (E) that all lack enamel deposition. Scale = 100μm. Abbreviations: **de**, dentine; **e**, enamel; **h**, heel; **os**, occlusal surface, likely formed of mantle dentine; **pc**, pulp cavity; **t**, talon.

Interestingly, we have been unable to find substantive enamel deposition along the heel of any developing teeth that were μCT scanned or thin-sectioned. For example, the developing incisor of NHCC LB370 was sectioned in a sagittal plane to examine deposition of tissues during crown formation but before eruption and occlusion (Fig 5A). On the lingual (occlusal) side of the tip of the talon a poorly preserved, but very thin (<19μm) layer of enamel is apparent (Fig 5B). This layer, however, extends cervically only briefly. The rest of the occlusal surface is formed by a thin (approximately 25μm) outer layer that can be differentiated from the dentine under polarized light by a granular band (Fig 5C–5E). Contrary to the distinctive structure of enamel apparent along the rest of this tooth, this layer most similar to mantle dentine, which forms regularly near the DEJ just deep to where enamel normally forms [40].

### Tooth attachment

Boonstra [23] was the first to note that tapinocephalid jaws were edentulous more frequently than those of other dinocephalians, which he postulated was related to differences in tooth attachment and replacement. Sidor [21] echoed these observations and broadened the comparison with other contemporaneous therapsids.

The tissues involved in tooth attachment in fossil vertebrates can be assessed by examining the microstructure of teeth and jawbone [49]. A fossil jaw with an ankylosis will display histological evidence of a connection between the bone forming the alveolus (alveolar bone) along the jaw and the cementum of the tooth root. A fossil jaw with a gomphosis will preserve alveolar bone, cementum lining the root of the tooth, and a periodontal space where the attaching ligaments once occupied. In fossils lacking soft tissue preservation, Sharpey’s fibers in both the alveolar bone and cementum are inferred to represent the mineralized fibers of a ligamentous connection between the two hard tissues, congruent with what has been described by LeBlanc et al. [33].

**Fig 6:**
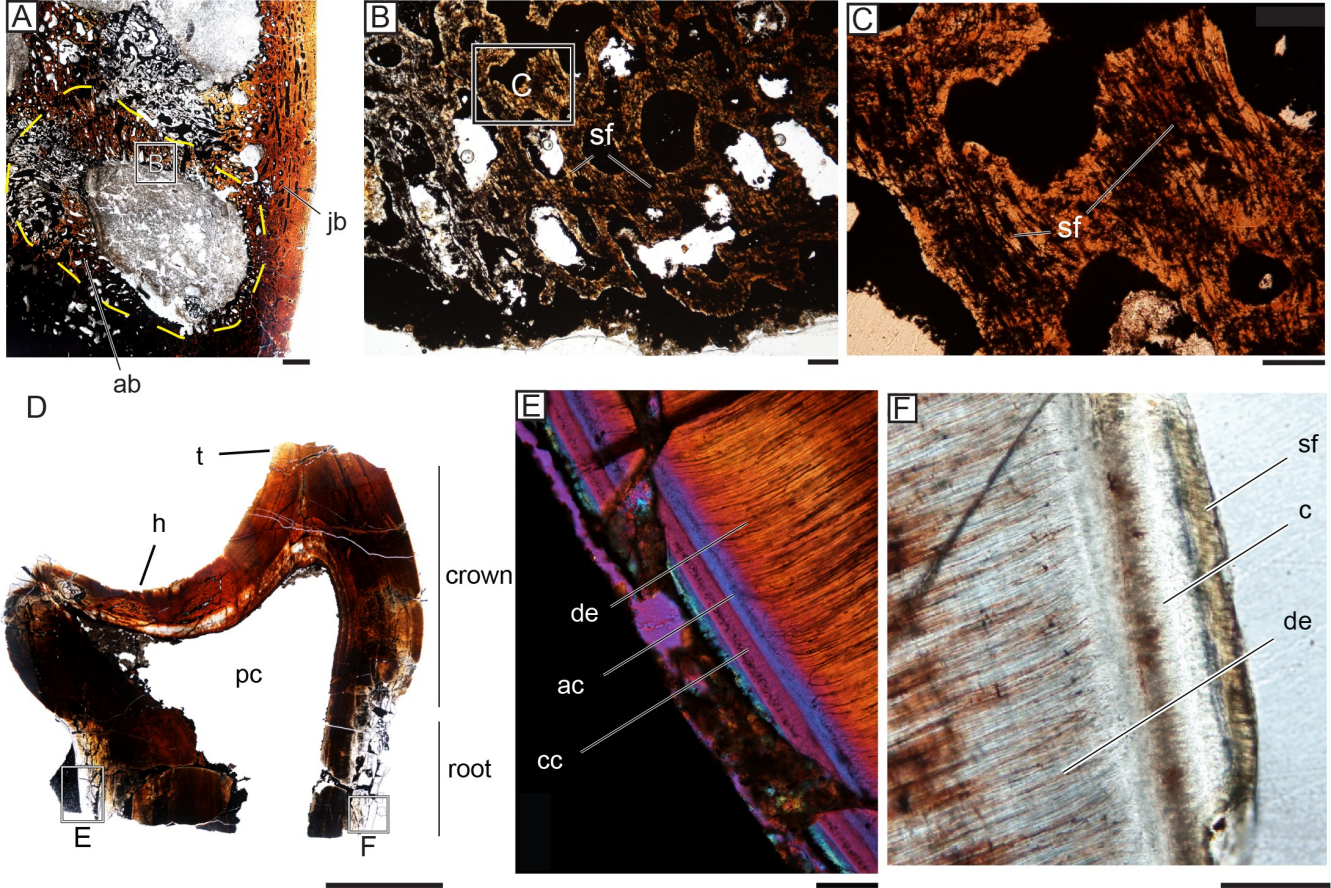
Evidence of a permanent gomphosis in tapinocephalid jaws and teeth. (A) Histology of NHCC LB369, an empty tooth socket with the alveolar bone outlined from jaw bone by yellow dashed line. Scale = 2000μm. (B) Alveolar bone from (A) with dense Sharpey’s fibers. Scale = 200μm. (C) Close up of Sharpey’s fibers from (B). Scale = 100μm. (D) Longitudinal thin section of NHCC LB733 that contains part of the root. Scale = 10000μm. (E) Close up of the lingual root of NHCC LB733 under polarized light with a lambda plate. Clear boundaries between the acellular and cellular cementum lining the tooth root are visible. Scale = 100μm. (F) Close up of the cementum of NHCC LB733 on the labial side of the root with Sharpey’s fibers under polarized light. Scale = 100μm. Abbreviations: **ab**, alveolar bone; **ac**, acellular cementum; **cc**, cellular cementum; **de**, dentine; **h**, heel; **jb**, jaw bone; **pc**, pulp cavity; **sf**, Sharpey’s fiber; **t**, talon.

Alveolar bone of NHCC LB369 and NHCC LB370 lines the empty tooth sockets and is characterized by woven bone that is highly vascularized (~58% vascularity) while the surrounding jaw bone is compact fibrolamellar bone with lower vascularization (~32%) (Fig 6A). Sharpey’s fibers are abundant in the alveolar bone and arranged roughly parallel to the apical-cervical axis of the root (Fig 6B and 6C), although the lack of teeth in situ makes it difficult to determine whether the ligaments acted as a sling or radiated from the alveolar bone.

The cementum of all three of the teeth that were sectioned is generally poorly preserved. The sections of cementum that are preserved have an average thickness of approximately 70μm, however it is likely that the outermost portion of the cementum is incomplete given its outer wavy border. Tapinocephalid cementum has two layers with an acellular layer found adjacent to dentine and a superficial cellular layer with cell lacunae (Fig 6E). Visible in the cementum are Sharpey’s fibers oriented perpendicular to the long axis of the tooth (Fig 6F).

Specimens available for sectioning did not include jaws with functional teeth to directly examine the relationship between the tooth roots, periodontal space, and alveolar bone. Unground serial sections of *Tapinocephalus* (SAM-PK-12139) preserve teeth in situ, and although the details of the tissues are not apparent, some positions preserve clear periodontal space surrounding the roots of functional teeth. Based on this specimen, we approximate the width of the periodontal space to be between 0.6mm and 1.7mm.

### Replacement

**Fig 7:**
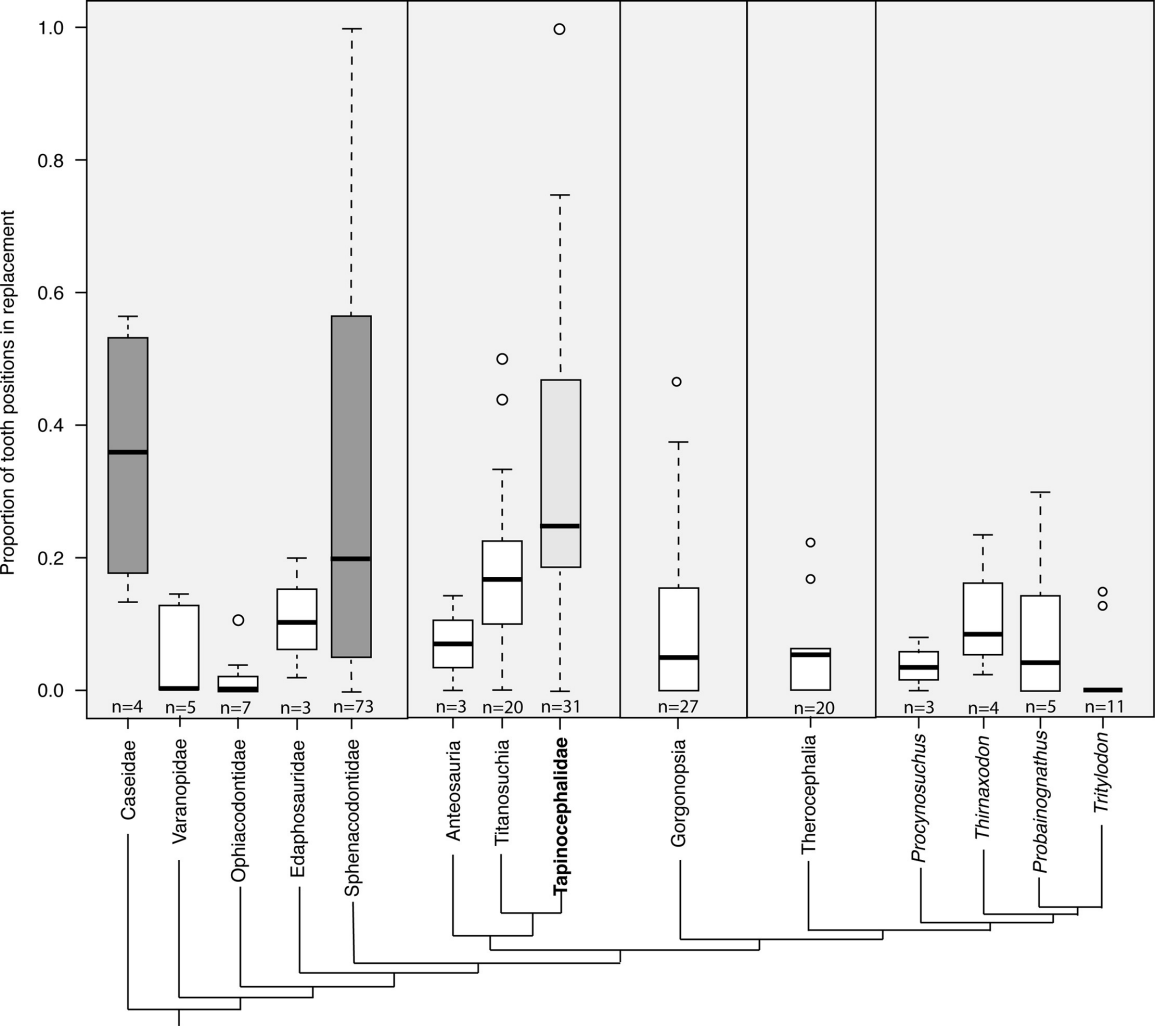
Proportion of tooth positions in replacement in fossil synapsids. Proportions are such that 0.0 represents no positions visibly in replacement and 1.0 represents all positions in visible replacement. With the exception of pelycosaurs and dinocephalians, synapsid clades generally have an average proportion below 0.1. Within pelycosaurs, caseids and sphenacodontids (dark grey boxes) have a significantly higher average proportion of teeth in replacement than do other pelycosaurs, and tapinocephalids (light grey box) have a higher average proportion than other dinocephalian clades. Caseids (*p =* 0.868) and sphenacodontids (*p =* 0.600) are the only groups to not have significantly fewer average number of teeth in replacement than tapinocephalids.

Teeth were actively being replaced in all 31 tapinocephalid jaws examined and comparative data show that tapinocephalids replace proportionally more tooth positions than other groups of therapsids (Fig 7). Furthermore, the inferred rapid rate of tooth replacement in tapinocephalids is most similar to that in basal synapsids, including carnivorous sphenacodontids and herbivorous caseids (Fig 7).

Although much attention has been paid to understanding patterns of replacement in non-mammalian cynodont therapsids (e.g. [50–52]) dinocephalian patterns of tooth replacement are limited to brief observations of titanosuchians recorded by Boonstra [23]. The adult *Tapinocephalus*, SAM-PK-12139, and the very small tapinocephalid, NHCC LB303, provide some insight into patterns of replacement although interpretations are limited due to the lack of a corresponding side and entire tooth row. These specimens suggest that tapinocephalid replacement was similar to other non-mammalian synapsids in that replacement was alternate and continuous through life (Fig 4F–4H).

## Discussion

### Specializations to herbivory

The tapinocephalid specimens included in this study provide evidence of a dentition adapted for the processing of plant material. Histological data including comparatively thick and wavy-like enamel, significantly reduced enamel deposition on the heel, subsequent wear of this surface, and a possibly wide periodontal ligament space, are congruent with previously described gross anatomical features indicative of herbivory.

#### Replacement

Herbivorous dentitions must adapt to the wear inflicted on teeth during the mechanical breakdown of plant matter. Alternating and rapid tooth replacement provides a mechanism to combat the wear tapinocephalid teeth encountered, providing a continuous source of unworn surfaces. Alternating pattern and frequent replacement, however, can result in periods of time where positions lack a functional tooth possibly interrupting precise occlusion and thus feeding. In tapinocephalids, the retention of a functional tooth late into a single position’s replacement likely mitigated this problem. Tapinocephalid teeth appear to have remained functional until replacement teeth were nearly erupted, minimizing the amount of time a tooth position was unavailable to assist with food processing. Evidence of this timing is apparent in the second tooth position of SAM-PK-12139, where replacement tooth is nearly entirely formed and in the functional position with the functional tooth not yet ejected (Fig 4F–4H). Retention of the functional tooth is likely aided by the lingual development and subsequent labial movement of the replacement tooth. In contrast to replacement that develops in the same position, labial drift of the replacement tooth allows the functional tooth to remain operative longer.

#### Gomphosis

Our findings note a particularly wide periodontal space and confirm the LeBlanc et al. [33] result of a permanent gomphosis in tapinocephalids. Gomphosis would have provided a mechanism to maintain occlusion in a dentition that that was rapidly replacing in addition to cushioning the forces of mastication. Although SAM-PK-12139 may represent an unusually wide periodontal ligament space (especially at its maximum of 1.7mm), this is much larger than the values reported for humans (0.15 to 0.38 mm [42]), crocodylians (0.37 mm [53]), and even animals with comparable body sizes like bovines (0.40 to 0.65 mm [54]). A wide ligament space would, at least in part, help to explain previous observations of tapinocephalid jaws frequently being recovered edentulous.

A possible explanation for the acquisition of a wide ligament may relate to the response of this ligament to force. The periodontal ligament is particularly adept at force perception which allows it to maintain occlusion through either tooth wear or jaw growth [55–56] as well as preventing damage to the tooth through cushioning and avoidance of unfavorable forces. The anchoring periodontal ligament of a gomphosis has been shown to widen with increased stress on teeth and jaws in modern mammals [57]. Thus, the acquisition of a permanent gomphosis in tapinocephalids could have been acquired in a response to occlusion while the maintenance of a wide ligament space could represent a response to the high, repeated forces encountered through increased oral food processing.

#### Enamel

Tapinocephalid enamel is strikingly similar to what has been reported for other grinding herbivorous dentitions in terms of its structure, development, and thickness (Table 2). A surprising finding of this study is the seemingly congenital lack of enamel on the heels of tapinocephalid incisors. Previous studies have noted this lack of enamel on the heel, but this absence has been interpreted as the result of wear during tooth-to-tooth occlusion (e.g.[29]). Our findings reveal that at least some tapinocephalids developed teeth without enamel on the heel and instead have an acellular tissue resembling mantle dentine capping the occlusal surface. Given the distribution of enamel around the rest of the tooth, it is most likely that the developmental shift resulting in this morphology was an initial formation of enamel-secreting ameloblasts, followed by an eventual termination of mineralization such that enamel did not form into its mineralized state. The lack of mineralization exclusively on the occlusal surface suggests an evolutionary modification akin to that seen in extant grazing mammals, in which ridges formed by enamel surround dentine valleys, thereby predisposing the tooth to wear into complex occlusal surfaces specialized for shearing and grinding [58–60].

**Table 2:** Selected herbivorous taxa highlighting details of their dentitions.

	Synapsida				Sauropsida			
					Diapsida			
						Dinosauria		
	^†^Tapinocephalidae	^†^*Diademodon*^[^^33^^,^ ^51^^,^ ^61^^]^	^†^*Cotylorynchus*^[^^33^^,^ ^51^^]^	*Equus*^[^^59^^,^ ^62^^]^	*Uromastyx*^[^^53^^]^	^†^*Iguanodon*^[^^44^^,^ ^46^^]^	^†^*Changchunsaurus*^[^^47^^]^	^†^*Captorhinus*^[^^44^^,^ ^63^^]^
**Enamel**								
max thickness (μm)	590	>100	>100	800	110	150	55	50
type	wavy-like	synapsid columnar	parallel crystallite	prismatic	prismatic	wavy	wavy	parallel crystallite
**Attachment**	gomphosis	gomphosis	ankylosis	gomphosis	ankylosis	gomphosis*	gomphosis	ankylosis
**Replacement**								
pattern	alternating	sequential	alternating	sequential	-	x	alternating	horizontal
# of events	polyphyodont	determinate polyphyodont	polyphyodont	diphyodont	monophyodont	polyphyodont	polyphyodont	polyphyodont
frequency of events	rapid	low	rapid	-	-	rapid	rapid	rapid
**Tooth Crown**								
morphology	expanded occlusal surface	expanded occlusal surface	peg-like	expanded occlusal surface	leaf-shaped	expanded occlusal surface	leaf-shaped	peg-like
regionalization	homodont	heterodont	homodont	heterodont	homodont	homodont	homodont	homodont
occlusion	yes	yes	no	yes	no	yes	yes	no
tissues in occlusion	dentine, enamel	enamel	enamel	dentine, enamel, cementum	enamel posteriorly, dentine anteriorly	dentine, enamel	dentine, enamel	enamel
other herbivory specializations	wide periodontal ligament space	x	palatal teeth	hypsodont	-	dental battery	-	Multiple marginal tooth rows; palatal teeth

^†^indicates extinct taxa

-, not applicable field; x, unknown field

*suggested character state.

A surprising result from these data is the presence of prismless wavy-like enamel in tapinocephalid teeth. Enamel that lacks even columnar structure is unexpected in tapinocephalids given the abundance of this feature in other therapsids. Synapsid columnar enamel (SCE) has been described in a variety of therapsid groups including anteosaur and titanosuchid dinocephalians [61]. Thus, the lack of columnar structure in tapinocephalids is, for the time being, best interpreted as being secondarily lost.

Furthermore, tapinocephalid enamel is potentially a remarkable case of convergence with previous descriptions of wavy enamel restricted to ornithopod dinosaurs [44, 46–47]. The lack of a wavy transmitted light pattern under cross-polarized light suggest caution in assigning this enamel type as wavy, however the crystallite structure does appear similar to previous descriptions of this enamel type and may have evolved under similar functional pressures. It has been proposed that wavy enamel may be an ornithopod specialization to shearing tough plant matter [45, 47], or resistance to crack propagation, analogous to the prisms of mammalian enamel [45]. In tapinocephalids, if wavy-structured enamel is considered in the context of the wide periodontal ligament and crushing heel morphology, this microstructural organization suggests it functioned in the prevention of crack propagation in the enamel. Yet, given the significant lack of enamel on the crushing heel, tapinocephalids were not subjecting their enamel to the repeated crushing forces suggested by Boonstra [64] and commonly used in descriptions [20, 27, 35, 66]. The lack of enamel on the basin of the heel however, does create an enamel ridge surrounding the heel that was likely useful in shearing plant matter. Thus, tapinocephalids may have evolved a specialized enamel to increase the efficiency of their food processing that allowed them to shear plant matter in addition to other adaptations that allowed them to crush their food.

#### Combination of traits

The data discussed here reveal an unexpected array of features in an early therapsid, providing an example of how descriptions of fossil taxa as having either a basal reptile-like or derived mammal-like dentition can be an oversimplification that masks surprising combinations of features. While polyphyodont replacement and prismless enamel are considered plesiomorphic traits, especially relative to mammalian dentitions, when the entire suite of traits comprising tapinocephalid dentition is considered, it is evident that these animals had a derived, alternative combination of features well-suited for herbivorous oral processing. The suite of features that comprise the tapinocephalid dentition are one combination of many that have evolved to enhance oral processing, often in taxa specialized for herbivory (Table 2). Individual traits do not operate in isolation and thus, the categorization of teeth as either “simple” or “complex” can be misleading about the specialization or effectiveness of an entire dentition. Furthermore, certain specializations may be more cryptic in fossil taxa (e.g. wavy enamel) and microanatomical analyses provide essential data that cannot be gleaned or can be incorrectly assigned from macroanatomical assessment alone.

#### Caveats

It is worth mentioning that the features discussed in this study are uniformly present within all sampled specimens. That said, we suspect some degree of variation within tapinocephalids likely exists. For instance, the tapinocephalid teeth included in this study have thicker enamel than previously reported for the tapinocephalid *Moschops* [65]. In addition, the present study is limited in two ways. First, the material available for thin-sectioning did not include features diagnosable to the genus-level, which limited our interpretations to Tapinocephalidae without narrower comparisons within the family. Second, the phylogeny of tapinocephalids is poorly resolved [66] and so it is unclear to what degree the features described here show taxonomically important systematic variation. A robust and detailed phylogeny for this clade with special attention to the characters of teeth and jaws would provide an important foundation upon which to make further conclusions about the evolution of dental features within Tapinocephalidae.

### The acquisition of derived dental features

The acquisition of mammalian features has often been described as a step-wise pattern (e.g. [21, 32]), including derived dental features (e.g. [52, 67]). Tapinocephalids independently evolved several mammal-like dental traits and can therefore act as a test of the sequence by which mammals acquired a complex dentition. Our results show that tapinocephalids are characterized by frequent, alternating, and continuous replacement as well as prismless enamel, all of which are plesiomorphic. This suite of plesiomorphies suggests that the acquisition of some derived mammal-like features (i.e. precise occlusion and permanent gomphosis) are not necessarily tied to the acquisition of a derived diphyodont replacement or prismatic enamel.

Furthermore, our data on tapinocephalids suggest that the continuous, rapid replacement of teeth may mitigate the selective pressures to evolve greater crack resistance in enamel structure, which has been hypothesized to be a factor in the evolution of prismatic enamel [68]. We suggest that continued sampling of fossil synapsid and sauropsid dentitions could reveal combinations of traits not previously considered, which can continue to test the relationships between traits as well as the sequence in which these traits were acquired in derived dentitions.

## Supporting information

S1 File Proportion of teeth in replacement of selected therapsid teeth. Data used to generate plot in Fig 7 with associated specimen numbers.
(CSV)
